# Vitamin D Review: The Low Hanging Fruit for Human Health

**DOI:** 10.1155/2021/6335681

**Published:** 2021-12-02

**Authors:** Lucy N. W. Mungai, Zanuba Mohammed, Michuki Maina, Omar Anjumanara

**Affiliations:** ^1^Department of Paediatrics, University of Nairobi, Nairobi, Kenya; ^2^KEMRI Wellcome Trust Research Programme, Kilifi, Kenya

## Abstract

Vitamin D is an important hormone that is known for the regulation of calcium and phosphate metabolism. Vitamin D deficiency leads to rickets in children and osteoporosis in adults leading to poor bone mineralisation and can also lead to serious dental complications in the same population. Recent studies have shown vitamin D to work as a hormone needed not only in bone and teeth but also in other body organs from intrauterine life up to old age. It has been demonstrated that Vitamin D has various effects on biological processes that deal with cell growth, differentiation, cell death, immune regulation, DNA stability, and neuronal growth. Despite being readily formed in the body through the intervention of the sun, patients are still found to have low vitamin D levels. We review studies done to show how vitamin D works.

## 1. Literature Review

### 1.1. Vitamin D, Source and Metabolism

Vitamin D is a hormone made up of a group of fat-soluble secosteroids whose primary known function is to increase intestinal absorption of calcium and phosphate. The most important compounds in this group are vitamin D_3_ (also known as cholecalciferol) and vitamin D_2_ (ergocalcifero l) [1]. In human beings, 90% of vitamin D comprises of cholecalciferol made through the skin, and 10% is ergocalciferol, ingested from the diet. The 7-dehydrocholesterol molecule, an intermediate metabolite in cholesterol synthesis found in the human skin, has a B ring that is broken by sunray irradiation through isomerisation to form secosteroid vitamin D_3_. This product is transported from the skin to the rest of the body, bound to vitamin D binding protein (DBP). Vitamin D_3_ is then metabolised in the liver by the hepatic vitamin D_3_-hydroxylase, a sterol 27-hydroxylase (CYP27A1) and vitamin D_3_ 25-hydroxylase (CYP2R1), to form 25-hydroxyvitamin D_3_, which is further hydrolysed in the proximal renal tubule to form 1,25(OH)_2_ D_3_ by 25-hydroxyvitamin D_3_ by 1*α*-hydroxylase (CYP27B1) [1].

The main active form of vitamin D is 1,25(OH)_2_ D_3_ and is tightly controlled by parathyroid hormone, phosphate, and fibroblast growth factor 23 (FGF23) [2]. There are other factors that control the extrarenal production of 1,25(OH)_2_ D_3_ (in the macrophages, osteoblasts, and keratinocytes), such as interferon-gamma, tumour necrosis factor, and interleukin 1 beta. High level of 1,25,(OH)_2_ D_3_ leads to its catabolism through the stimulating CYP24A1, an enzyme that increases the inactive 24,25,(OH)_2_ D and 1,24,25,(OH)_3_ production. Vitamin D is transported in blood bound to albumin and vitamin D binding proteins, but when there is reduced protein synthesis in the liver or increased loss of protein in the gut or kidney the free serum vitamin remains normal but total vitamin D is low [1].

### 1.2. The Role of Vitamin D in General Body Growth and Development

Several studies have suggested an association between intrauterine life events and being susceptible to disease in adult life in a process called foetal programming or developmental origins of health and diseases [3]. Vitamin D has been shown to have a role in foetal implantation, tolerance and placental development. The potent immunosuppressive effects of 1,25(OH) D_3_ indicate its function in preventing preeclampsia in pregnancy [4]. Researchers postulated that, as a result of such events in utero environment, foetal programming induces specific genes and genomic pathways that control foetal development and subsequent disease risk [5]. Therefore, prenatal vitamin D deficiency may not only affect maternal skeletal preservation and foetal skeletal formation but also influence foetal “imprinting” that may affect chronic disease susceptibility postnatally [3].

Vitamin D regulates cell proliferation and differentiation and can protect the genome from daily life insults such as oxidative stress and toxins. Treatment with vitamin D has been shown to decrease 8-hydroxy-2-deoxyguanosine, a marker of endogenous oxidative damage to DNA [3].

### 1.3. Vitamin D and Chronic Conditions

Epidemiological and some randomised controlled studies link hypovitaminosis D with diseases such as high blood pressure, muscular disorders, propensity to get infection, autoimmune disorders, and malignancy. Generally, 1, 25, vitamin D_3_ has anticancer properties that are of possible clinical importance. It inhibits clonal proliferation of leukaemic cells. Through the interaction between 1,25 Vit D and vitamin D receptor (VDR) complex, it inhibits the malignant cell cycle at G1-GO transition. It induces apoptosis which contributes markedly to growth suppression properties of sterols in hyperproliferative disorders. In breast cancer cells, 1,25(OH)_2_ D_3_ induces apoptosis through reciprocal modulation in Bc12 and Bax content which promotes the death of cancer cells. It increases intracellular calcium, which activates the calcium-dependent proapoptotic proteases, microcalpain, and caspase 12. It also enhances the antitumoral properties of ionising radiation [6]. Vitamin D has been shown to have other nongenomic action such as antiproliferative properties on keratinocytes in skin diseases such as psoriasis and immunosuppressive properties on autoimmune destruction of beta cells to prevent type I diabetes mellitus. Vitamin D is now in use for the treatment of psoriasis [7].

Epidemiological studies have associated inadequate vitamin D with hypertension and high plasma renin activity. In VDR null mice, marked elevation of renin expression and plasma angiotensin II production lead to hypertension, cardiac hypertrophy, and increased water intake [3]. When these animals were supplemented with vitamin D, renin production was suppressed.

In other experimental animals, vitamin D deficiency was associated with an earlier and more aggressive form of diabetes, probably related to abnormalities in immune function and impaired glucose tolerance. Through a VDR-mediated modulation of calbindin expression, vitamin D controls intracellular calcium flux in islet cells, which in turn affects insulin secretion. 1,25(OH)_2_ D_3_ deficiency, found in chronic renal failure, results in disorders of insulin secretion and blunted response of pancreatic beta cells. Karau et al. in their vitamin D prevalence study among diabetes patients found low vitamin D levels in about 57% of the participants [8]. Supplementation with 1,25(OH)_2_ D_3_ has been shown to correct abnormal insulin secretion independently of changes in serum levels of calcium or parathyroid hormone (PTH) [9].

Evidence supporting direct action of 1,25(OH)_2_ D_3_ on skeletal muscles growth and differentiation is known. Vitamin D has been found to have a dual effect on the musculoskeletal system. It improves bone mass density and muscle mass, strength, and function. In addition, sufficient vitamin D status has been shown to reduce the risk of falling in older individuals, most likely by improving neuromuscular functions. This is evident in chronic renal failure patients who present with weakness and atrophy related with 1,25(OH)_2_ D_3_ deficiency [2, 10].

### 1.4. Reproduction

Calcium is needed in the male reproductive tract, specifically spermatogenesis, sperm motility, hyperactivation, and acrosome reaction. A population study involving 300 men indicated that men with hypovitaminosis D had a lower proportion of motile, progressive motile, and morphologically normal sperms than men with normal vitamin D status. Vitamin D (1,25(OH)_2_ D_3_) increased calcium concentration in human sperm cells in vitro through VDR-mediated calcium release, improved sperm motility, and stimulated the acrosome reaction in vitro. Data from the LURIC study involving 2069 coronary angiography male patients suggest that the presence of both low vitamin D and low testosterone was associated with a 2.5-fold increased risk of dying, compared to men with a sufficient vitamin D and testosterone level [11].

### 1.5. The Role of Vitamin D in Bone and Tooth Development

There are six stages of tooth development: initiation stage, bud stage, cap stage, bell stage, apposition stage and maturation stage (crown stage), or calcification stage. Enamel formation by the ameloblast cells or a process commonly referred to as amelogenesis begins during the crown stage. Human enamel forms at around the third or fourth month of intrauterine life [12]. Amelogenesis can be divided into two stages. The first stage is called the secretory stage, which involves proteins and organic matrix forming a partially mineralised enamel. During this stage, the enamel proteins are released into the surrounding relevant areas of the jaws and contribute to what is known as the enamel matrix, which is then partially mineralised by the enzyme alkaline phosphatase [13]. The matrix eventually becomes an enamel rod, and the walls become interrod enamel. The second stage is the maturation stage, which completes the enamel mineralisation. In this stage, the ameloblasts transport substances used in the formation of enamel. They change their function from production, as in the secretory stage, to transportation. Proteins used for the final mineralisation process come from transported material. By the end of this stage, the enamel becomes completely mineralised.

Calcium is an essential constituent element in the mineralised enamel and is a component of enamel crystals at several levels. Extracellular Ca^2+^ is critical for the precipitation of ions from fluid and is required for enamel crystal growth [14]. The physiologic involvement of Ca^2+^ in the enamel formation is also evident by the presence of enamel defects in vitamin D-deficient animals. The deficiency results from the disruption of the normal plasma Ca^2+^ concentration resulting in hypocalcemia [15]. It has been reported that the main calcium influx pathway involved in the mineralisation of enamel, called the Ca^2+^ release-activated Ca^2+^ channel (CRAC), is critical for controlling calcium uptake and necessary for the development of the tooth enamel. Calcium is stored in the endoplasmic reticulum (ER) of cells until it is needed. In many cells, the protein STIM1 acts as a sensor in the ER, ensuring the right balance of calcium inside the ER and raising the alarm when calcium levels are low.

Stromal interacting molecule 1 (STIM1) plays a role in mediating store-operated Ca^2+^ entry (SOCE), a Ca^2+^ influx following depletion of intracellular Ca^2+^, and acts as a Ca^2+^ sensor in the endoplasmic reticulum. Upon calcium depletion, it translocates from the endoplasmic reticulum to the plasma membrane, activating the Ca^2+^ release-activated Ca^2+^ channel (CRAC) subunit. Stromal molecule 1 (STIM1) interacts with the Ca^2+^ release-activated Ca^2+^ channel in the cell membrane, allowing calcium from the blood into the cell to restore balance.

The mineralisation of the crown of primary teeth starts from the 3^rd^ intrauterine month till 12 months after birth, while mineralisation of permanent teeth starts from birth to 8 years, except the 3rd molars, which goes on until 16 years of age. Therefore, any disruption of this process by disease or deficiency leads to poor mineralisation of the teeth. [12].

The effect of calcium deficiency on tooth and tooth development has been seen in rickets patients. Studies have shown that vitamin D deficiency rickets presents itself before two years, while rickets due to calcium deficiency presents after two years of age. Patients with rickets tend to have increased alkaline phosphatase levels which might interfere with the transition from bud to cap stage of tooth development. This has been evident in patients with hereditary vitamin D-dependent ricket, hereditary, vitamin D-resistant rickets, and hypophosphatemic rickets. These defective teeth are more prone to caries because of poor mineralisation. There is also the risk of increased structural developmental defects (lamellae) which lead to early bacterial infection [12].

Vitamin D is an important requirement in various physiological processes, including bone and calcium metabolism, cellular growth, differentiation, and immunity. The widely known physiologic functions of vitamin D are the maintenance of serum calcium and phosphorus levels within the normal range for various metabolic functions such as transcription regulation and bone metabolism. Through the interaction with vitamin D receptor (VDR) in the small intestine, 1,25(OH)_2_ D_3_ increases the intestinal calcium and phosphorous absorption by approximately 30% and 20%, respectively, while interacting with VDR in osteoblasts to stimulate a receptor activator of nuclear factor kappa B ligand on immature preosteoclasts. This process then stimulates the osteoclasts to become mature bone-resorbing osteoclasts. The mature osteoclast removes calcium and phosphorus from the bone to maintain blood calcium and phosphorus levels, and in the kidneys, 1,25(OH)_2_ D stimulates calcium reabsorption from the glomerular filtrate [12].

### 1.6. The Role of Vitamin D in Dental Caries and Periodontitis

Dental caries: dental caries occur due to the demineralisation of enamel and dentine (the hard tissues of the teeth) by organic acids formed by bacteria resident within the dental plaque through the metabolism of sugars and other fermentable carbohydrates present in the plaque, resulting in the formation of organic acids that increase the solubility of calcium hydroxyapatite in the dental hard tissues leading to demineralisation of the hard tissues of the tooth. Deficiencies of vitamin D, vitamin A, and protein energy malnutrition (PEM) have been associated with enamel hypoplasia. Posteruptively, this condition predisposes the affected tooth to dental caries and even periodontal disease. PEM and vitamin A deficiency are also associated with salivary gland atrophy, which subsequently can lead to less saliva in the mouth, predisposing the teeth to dental caries and periodontal disease. Malnutrition, particularly the lack of protein and deficiencies of certain micronutrients such as vitamins, zinc, and iron, can influence the amount and composition of saliva, limiting the protective effects it has in the oral cavity. Undernutrition coupled with daily increased amount and/or frequency of sugars results in levels of caries greater than expected for the level of sugars intake [13–17].

The results of the National Oral Health Survey of Kenya 2015 showed the prevalence of dental caries in Kenyan children to be 23.9%, with the number of teeth affected with caries ranging from 1–14 teeth. This prevalence of dental caries was higher among the 5 year olds (46.3%) when compared to the other age groups. In the adult population, the prevalence of dental caries was 34.3% and 0.4% adults had missing teeth due to caries. It is, therefore, a common disease in adults and children in Kenya [18, 19].

Periodontal disease or gum disease is a pathological inflammatory condition of the gum and bone surrounding the teeth. The two most common periodontal diseases are gingivitis and periodontitis. Gingivitis is the inflammation of the gum at the neck of the teeth, while periodontitis is the inflammation affecting the bone and tissues of the teeth. The two forms of periodontal disease can be acute or chronic as well. The rate of progression of periodontal disease can depend on the virulence (or strength of attack), of bacteria in the dental plaque and the efficiency of the local and systemic immune-inflammatory responses in the host. Current research works have suggested that specific environmental and genetic factors can influence host responses, which determine the general susceptibility of that host or the local susceptibility of the supporting tooth tissues to periodontal disease.

The National Oral Health Survey of Kenya 2015 indicated that the prevalence of gingival bleeding among children was 75.7%, with children aged five years having the highest (99.6%) prevalence of gingival bleeding. In adults, gingival inflammation was found in 98.1%, with those in the age range 35–44 years having a 96.1% prevalence and those above 60 years having up to 99.3% prevalence of bleeding gums. [19] Though the participants did not check their serum vitamin D in this study, Karau et al. in 2019 found the prevalence of low vitamin D among Kenyan diabetic patients to be at 60%. This indicates that despite Kenya having sufficient sunlight almost throughout the year, low vitamin D is still prevalent.

Low vitamin D leads to the formation of osteopenic bone that is characterised by the loss of both the organic and mineral contents of bone throughout the body, including the maxilla and the mandible. The lowered density in the jawbone leads to increased alveolar porosity, altered trabecular pattern, and more rapid alveolar bone resorption followed invasion by periodontal pathogens. Periodontal infection increases the systemic release of proinflammatory cytokines which accelerate systemic bone resorption [20–26].

Recent studies have reported a high prevalence of vitamin D deficiency in the general population [6]. In these studies, women have vitamin D deficiency, thus requiring supplements during pregnancy and breastfeeding [27]. It has been reported that sufficient vitamin D level in pregnancy is important for foetal bone formation, tooth development, and possibly foetal growth and development [27]. Maternal vitamin D level has long-term effects on the teeth of the baby. [28].

The evidence that has so far been gathered on vitamin D demonstrated that vitamin D deficiency might predispose subjects at risk to low mineral bone density or osteoporosis and osteopenia and infectious and chronic inflammatory diseases [13, 19–22]. Through the direct and indirect effects of vitamin D on bone and mineral metabolism, innate immunity, and several vitamin D receptor gene polymorphisms, vitamin D has been associated with periodontal diseases [23]. Furthermore, Schroth et al., who assessed the relationship between vitamin D status and dental caries in 6 11-year-old Canadian school children, found 56.4% of children had dental caries whose presence was significantly associated with low vitamin D, poor oral hygiene, and low level of education [6].

## 2. Vitamin D and Immunity

Innate immunity is a nonspecific form of immunity and has a role of sensing pathogens through pathogen-recognition receptors (PRR) which lead to a cascade of events that end up with inactivation of microorganisms by stimulation of toll-like receptors (TLRs). The adaptive immunity is a specific form of immunity and occurs with antigen presentation by dendritic cells to the antigen recognition B and T lymphocytes that led to secretion of cytokines and killing of microorganisms. Studies show that innate immunity which is channelled through dendritic cells, macrophages, monocytes, and *T* and B cells can be regulated by vitamin D. Vitamin D has been shown to stimulate the maturation of anti-inflammatory cytokines and inhibit production of proinflammatory cytokines [29–31].

Vitamin D activates the innate immunity by increasing macrophage phagocytosis and inhibits the production of interleukin-10 (IL-10) and proinflammatory cytokines [20]. The active 1*α*,25(OH)_2_ Vit D strengthens barrier function at the epithelium by upregulating gene expression for gap junction, adherence, and tight junction proteins [32–34] Vitamin D regulates the proinflammatory/anti-inflammatory balance promoting an anti-inflammatory clinical manifestation of diseases to avoid tissue damage [34].

Studies have shown vitamin D deficiency increases the risk of several infections such as human immune deficiency virus, COVID-19, influenza, and tuberculosis. Supplementation with vitamin D in patients with these conditions is indicated to reduce the risk of developing the infection, reduce the severity, or prevent serious complications [35–38].

### 2.1. Vitamin D and Nonalcohol Fatty Liver Disease (NAFLD)

Finally, in the last decade, research has found a coexistence of vitamin D deficiency and nonalcohol fatty liver decease (NAFLD) [5]. This is a condition that leads to the accumulation of lipids in the liver in the form of triglycerides and elevated liver enzymes mainly alanine aminotransferase and aspartate aminotransferase with no excess alcohol consumption. Though NAFLD has been found in children, adolescents, and adults, it is a more common pathologic condition in elderly women with vitamin D deficiency and is considered the hepatic manifestation of the metabolic syndrome. The global prevalence of NAFLD is estimated at 25% [39, 40].

Though there is no registered treatment for NAFLD, there are some interventions that seem to improve the condition. Hoseini et al. did eight weeks of intervention study on 40 women between 60–65 years old with NAFLD. They divided the women into 4 groups: vitamin d and aerobic training (AT), aerobic training, vitamin D, and control on placebo and sedentary life style. Fatty liver reduced by 60% on the group on AT and vitamin D and the group on vitamin D only reduced by 22%. The combination of AT + vitamin D significantly reduced liver enzymes, anthropometric indices, and glycemic indices and improved lipid profile. The group on sedentary life style and placebo had increased fatty liver by 17.6%. They concluded that an adequate level of plasma vitamin D was necessary to achieve the beneficial metabolic effects of aerobic training [41]. On the other hand, the meta-analysis by Jaruvongvanich et al. that looked at the association between the severity of NAFLD histology and serum vitamin D showed no relationship [42]. This was similar to Wei et al.'s meta-analysis study where they explored the effects of Vit D supplementation in patients with NAFLD. They concluded that vitamin D supplementation neither improved insulin resistance nor liver enzymes and lipid profiles [43]. This is an area that requires further research (Table 1).

## 3. Conclusion

Studies have shown vitamin D is an essential vitamin needed for body processes and metabolism. There are scanty longitudinal studies conducted in countries along the equator that can lead to international guidelines on sun exposure. This would also lead to policies on building architectural plans, especially in developing countries that allow workers to have sun exposure during working hours. It is our low hanging fruit provided by mother nature.

## Figures and Tables

**Table 1:** The role of vitamin D in different body systems.

Body system	Role of vitamin D
General body growth and development	It is associated with intrauterine foetal programming.
	It plays a role in preventing preeclampsia in pregnancy.
	It regulates cell proliferation and differentiation and can protect the genome from daily life insults such as oxidative stress and toxins
Vitamin D and chronic conditions	It has been shown to inhibit clonal proliferation of leukaemic cells.
	Vitamin D has antiproliferative properties that are protective against chronic diseases such as psoriasis and type 1 diabetes mellitus.
	Low vitamin D has been associated with increased insulin resistance leading to type 2 diabetes mellitus.
	It has been shown to reduce the risk of falling in older individuals, especially those with chronic renal failure.
Vitamin D and reproduction	It improves spermatogenesis, sperm motility, and acrosome activation.
	Low vitamin D and low testosterone are associated with a 2.5-fold increased risk of dying in cardiac male patients.
Vitamin D in bone and tooth development	Low vitamin D is associated with poor intrauterine mineralisation of the crown of primary teeth and poor mineralisation of permanent teeth postnatally.
	Low vitamin D leads to rickets in children and osteoporosis in adults.
Vitamin D in dental caries and periodontitis	Low vitamin is associated with low calcium leading to dental caries. It also predisposes these patients to have periodontal disease.
Vitamin D and immunity	It increases macrophage phagocytosis.
	Vitamin D augments stimulation of the maturation of anti-inflammatory cytokines and alleviates production of proinflammatory cytokines.
	Vitamin D deficiency increases the risk of several infections. Supplementation with vitamin D reduces the risk of developing infection, reduces the severity, or prevents serious complications.
Vitamin D and liver	Vitamin D deficiency is associated with nonalcohol fatty liver disease (NAFLD)

## Data Availability

No data were used to support this review paper.
